# Public awareness of low vision rehabilitation in China

**DOI:** 10.3389/fmed.2025.1659439

**Published:** 2025-08-22

**Authors:** Xiaoman Li, Ruyi Zhu, Yuanyuan Liu, Jianing Zhang, Xiaoyue Hu, Qi Chi, Na Lin, Jie Chen

**Affiliations:** ^1^The Center of Excellence in Low vision and Vision Rehabilitation, Eye Hospital of Wenzhou Medical University, Wenzhou, China; ^2^National Clinical Research Center for Ocular Diseases, Wenzhou, Zhejiang, China; ^3^The Optometry Center, The Quzhou Affiliated Hospital of Wenzhou Medical University, Quzhou People's Hospital, Quzhou, Zhejiang, China

**Keywords:** low vision rehabilitation, awareness, questionnaire, vision professionals, public

## Abstract

**Objective:**

To demonstrate public awareness of low vision rehabilitation (LVR) in China and identify related influencing factors.

**Methods:**

This cross-sectional study assessed low vision rehabilitation awareness using a Delphi method validated questionnaire. The questionnaires were distributed through *Vision China* conferences (2022–2023) and representative hospitals in all provinces across China, targeting public and vision professionals.

**Results:**

1,482 questionnaires (360 were from visually impaired individuals) were collected, including 952 from the public and 530 from vision professionals. The mean total scores accounted for 37.0% and 55.6% of the maximum scores, respectively. For the public, individuals with higher education, good visual acuity, working in medical system, and having eye check yearly or more frequent were associated with better awareness of LVR. For vision professionals, participants aged over 40 years old, with better visual acuity, and knowing the low vision referral process demonstrated a better awareness of LVR. The main sources of LVR information for the public were doctors' advice (about 29%) and news media/online information (about 28%).

**Discussion:**

The awareness of LVR in China is insufficient in both the public and vision professionals. Regular eye checks, doctors' advice, and scientific education are essential to improve the awareness of the public. It is crucial to develop low-vision education in the medical curriculum and expand low-vision continuing education and forums to improve awareness among vision professionals.

## Introduction

The World Health Organization (WHO) adopts visual acuity in daily life as the criterion for assessing visual impairment, categorizing it into mild, moderate, severe visual impairment, and blindness. Low vision is categorized into moderate and severe visual impairment (1). According to the 2020 WHO vision report, 2.2 billion people in the world were visually impaired (2), among which 295 million were moderate to severe visual impairment (3). People with low vision usually have a decreased independence and quality of life (2, 4, 5). Low vision rehabilitation (LVR) could minimize the impact and improve the quality of life (6–8). Application of low vision aids, functional visual acuity training, skills training and psychological supports are all efficient methods for LVR (2, 4).

Besides the global initiative for the elimination of avoidable blindness, China Disabled Persons' Federation (CDPF) and National Health Commission of China have done lots of work to promote LVR services in China (9). The 13th 5-Year Plan of National Eye Health, released by National Health Commission of China, has stipulated that tertiary hospitals must set up low vision clinic and provide LVR services (10). With the efforts of the National Health Commission of China and CDPF, LVR services have been gradually improved during the past decades (11).

However, considering the large number of low vision population and relatively few vision professionals in China, only a very low proportion of people with low vision are actually receiving LVR services (12). One of the reasons is that the amount of ophthalmologists and optometrists is far less than enough. Another reason is suspected that the accessibility and availability of LVR services is not fully opened to the public. Many of the low-vision patients regretted not going to LVR clinics earlier, and many others refused to accept rehabilitation because of misunderstandings. Previous researches have revealed that the acceptance rate of low vision aids among elderly patients with visual impairment in Wenzhou, China was below 50%. Around 60% of the individuals with visual impairment still prefer to conventional refractive correction to improve their vision (13, 14).

The insufficient access and low acceptance of LVR in China demonstrated that cognitive blind spots or misunderstandings LVR exist in the public. With this in mind, this study was designed and conducted to demonstrate the current state and related factors of LVR awareness of the public in China. Then scientific education as well as low-vision training for the vision professionals would be targeted designed to improve the present situation.

## Methods

### Questionnaire design

A “Survey of public Awareness on Low Vision Rehabilitation in China” questionnaire was designed and used to demonstrate the current state of LVR awareness. There were two subscales of awareness items in the questionnaire, which was the public subscale and vision professional subscale, respectively. If the interviewee was a vision professional, including ophthalmologists, optometrists, eye nurses, vision therapists, and vision company practitioners, then they answered items for vision professionals, otherwise they answered items facing to the public. An additional part of the questionnaire was items designed for people with visual impairments. This part aimed to reveal their situation on visual rehabilitation services.

The Delphi method (15–18) was used to select items and test the effectiveness and reliability of the questionnaire. An item bank (57 items) was collected through literature review and professional experiences. Two rounds of expert consultations were conducted based on the item bank. The expert consultations involved 20 experts, including seven low vision specialists, one rehabilitation training professional, eight ophthalmologists, three statistics experts, and one special education expert. One ophthalmologist's advice was not reliable and was excluded. After two rounds of expert consultations and a pilot study, a finalized questionnaire was produced, which included eight demographic items for all participants, 10 for the public, 16 for the vision professionals, and four for the visually impaired (Appendix 1). The Cronbach's α is 0.7 in vision and non-vision professional items.

### Survey and data collection

An online mobile application named Wenjuanxing, which has functions equivalent to Amazon Mechanical Turk, was used to distribute the questionnaire. Individuals scan a QR code to access and answer the questionnaire.

This survey was conducted with two distinct participant groups: the general public and vision professionals. The questionnaire was distributed through representative hospitals in all provinces across China. Additionally, the survey was distributed to vision professionals attending the *Vision China Conference* in 2022 and 2023. *Vision China Conference* is one of the large-scale visual conferences in China. Attendees include ophthalmologists, optometrists, nurses, researchers, and visual enterprise employees, representing a highly representative cohort in vision professionals.

The sum score of all items of each participant was used to determine the person's LVR awareness. The mean score of an item of all the participants was used to assess the awareness of a particular item. If all the items were answered correctly, a maximizing score would be obtained.

### Statistical method

Data was analyzed using a Chinese version of SPSS 25.0. Mean ± SD and median (quantile) were used to describe quantitative data. Frequency and composition ratios were used to describe categorical data. The sum score of each subscale was calculated. Principal component analysis (PCA) was used for questionnaire structure analysis. One-way analysis of variance (ANOVA) and logistic regressions were performed to show the relations of scores with demographics. *p* < 0.05 were considered as statistically significant.

## Results

### Demographic information

A total of 1,482 questionnaires were collected, among which 952 was from the public and 530 was from the vision professionals. Six vision professional participants were excluded because they were < 18 years old, so 524 valid vision professional questionnaires were analyzed (Table 1).

**Table 1:** Demographic information of the participants.

**Factors**	**The public**		**Vision professionals**		**Factors**	***N***	**(%)**
	***N***	**(%)**	***N***	**(%)**			
**Age**					**Occupation of the public**		
≤40 years	701	73.6	430	82.1	Without medical background	579	60.8
>40 years	251	26.4	94	17.9	With medical background (vision not included)	373	39.2
**Gender**					**Occupation of vision professionals**		
Male	320	33.6	139	26.5	Ophthalmologist	147	28.1
Female	632	66.4	385	73.5	Optometrist	137	26.1
**Education**					Visual technicians, assistants, and nurses	157	30.0
High school or below	111	11.7	0	0	Other vision-related professionals	83	15.8
Vocational school	161	16.9	97	18.5	**Characteristics of institutions where vision professionals work in**		
Bachelor's degree	478	50.2	251	47.9	**(1) Hospital/rehabilitation institution level**		
Master's degree or above	202	21.2	176	33.6	Third-grade^**^	275	59.5
**Area**					Others	187	40.5
Rural and suburban area	273	28.7	60	11.5	**(2) Presence of low vision center/visual rehabilitation department**		
Urban	679	71.3	464	88.5	No, no intention to establish	43	9.2
**Self-reported PVA** ^*^					No, but intend to establish one	86	18.4
Poor vision	105	11.0	28	5.3	Yes, immature conditions/technology	94	20.1
Moderate vision	165	17.3	61	11.6	Yes, mature conditions/technology	245	52.4
Good vision	682	71.6	435	83.0	**(3) Presence of low vision rehabilitation referral process**		
**Frequency of eye check**					No/do not know if there is a referral process	145	31.2
Never	100	10.5	21	4.0	Yes	320	68.8
Only when needed	439	46.1	185	35.3			
Interval >1 year	188	19.7	84	16.0			
Interval ≤ 1 year	225	23.6	234	44.7			

* Self-reported PVA stands for presented visual acuity of the better eye in daily life reported by the research participants themselves.

** Third grade stands for the top hospital level in China authorized by the health commission of China.

**Figure 1:**
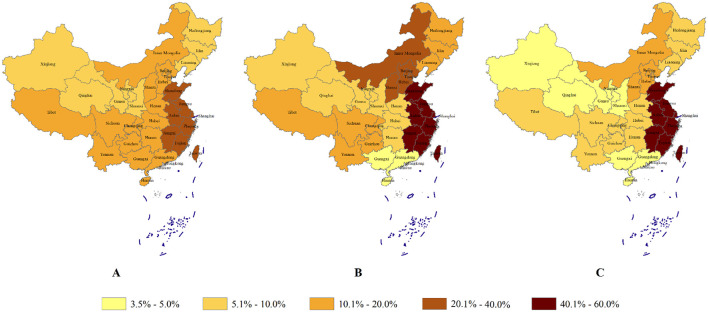
The proportion of population in each region of China and the proportion of the the questionnaire subjects in each region of China. Three maps of China labeled A, B, and C show varying population percentages. Color gradients: light yellow represents 3.5% to 5%, increasing to dark brown for 40.1% to 60%. Map A is the real proportion of populations in each region of China. Map B is the proportion of subjects who answered the public items in each region. Map C is the proportion of subjects who answered the vision professionals items in each region of China.

**Figure 2:**
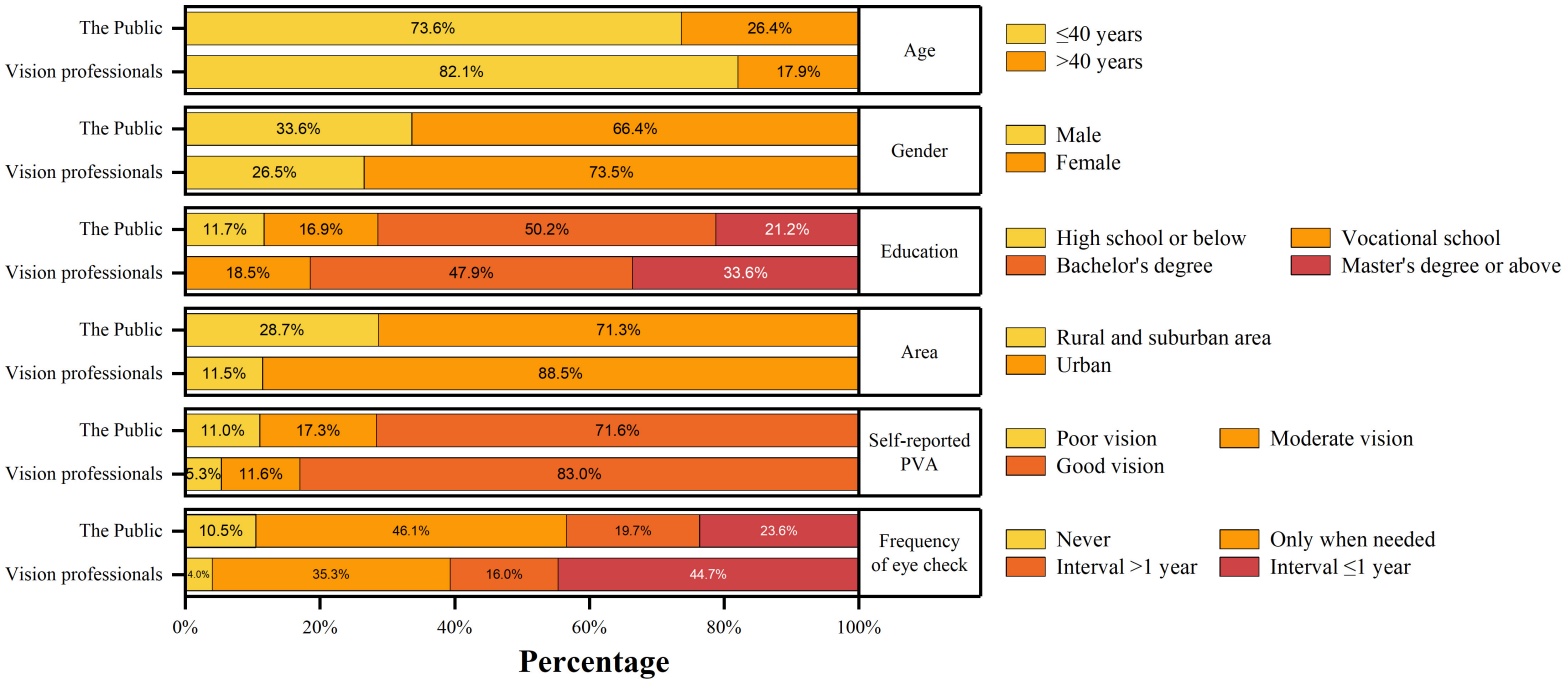
Demographic information of the participants.

Regardless of the public and the vision professionals, the majority of the participants was from Eastern China (57.9% in the public; 48.2% in the vision professionals, Figure 1). The age, gender, education level proportion and visual acuity was all similar in both groups. About 70% of the public and 90% of the vision professionals lived in urban areas, respectively. Details were listed in Table 1 and Figure 2.

**Figure 3:**
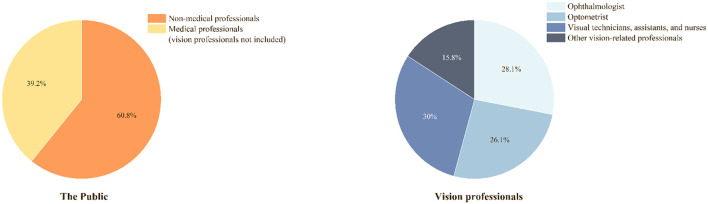
Classifications of the public and the vision professionals.

The majority of the public participants were without medical education background (60.8%). For vision professional participants, 59.5% worked in a tertiary hospital/institute, and 68.8% knew how to refer low-vision patients to LVR departments (Table 1, Figure 3).

### Sum score of LVR and principal component analysis

The mean sum score of all items in the public group accounted for 37.0% of the maximizing score (17.4/47.0). All the items could be categorized into 3 principal components, which accounted for 71% of the total variations, using principal component analysis (PCA). The three components were interpreted as *awareness of LVR* (PC1), *awareness of low vision* (PC2), *understanding of the current living status of low vision population* (PC3). The percentage of the mean score of the three principal components accounted for 31.0, 58.1 and 32.1% of the maximizing score, respectively (Table 2).

**Table 2:** Item scores for the public.

**Principal component (PC, percentage of variance %)**	**Maximal score (MS)**	**Mean ±SD**	**Mean/MS (%)**
**PC1: awareness of low vision rehabilitation (40%)**	28	8.7 ± 6.7	31.0
How much do you know about low vision rehabilitation?	3	1.0 ± 0.9	34.0
Which of the following methods belong to low vision rehabilitation?	7	2.5 ± 2.2	35.0
Which places provide low vision rehabilitation services?	7	2.1 ± 1.9	29.7
Would low vision rehabilitation improve individuals' quality of life?	3	1.4 ± 1.0	45.0
Are you aware of the subsidy policies provided by China disabled persons federation?	3	0.6 ± 0.9	20.0
Where do you get low vision rehabilitation-related knowledge?	5	1.2 ± 1.1	23.8
**PC2: awareness of low vision (18%)**	10	5.8 ± 2.6	58.1
What aspects would be affected by low vision?	7	4.3 ± 2.0	61.2
Which of the followings are considered as low vision?	3	1.5 ± 1.0	49.7
**PC3: understanding of the current status of low vision population (13%)**	9	2.9 ± 1.3	32.1
Where are children with severe visual impairment usually educated at?	3	1.0 ± 0.6	33.3
What do individuals with severe visual impairment usually do for a living?	6	1.9 ± 1.0	31.7
**Cumulative (71%)**			
Total	47	17.4 ± 8.4	37.0

The mean sum score of all items of the vision professionals accounted for 55.6% of the maximizing score (32.3/58.0). All the items were also categorized into three principal components, which accounted for 60% of the total variations, using PCA. The 3 components were interpreted as *Detailed information of LVR* (PC1), *Overall impression of LVR* (PC2), *understanding of the current living status of low vision population* (PC3). The percentage of the mean score of the three principal components accounts for 59.5, 57.8 and 36.0% of the maximizing score, respectively (Table 3).

**Table 3:** Item scores for the vision professionals.

**Principal component (PC, percentage of variance %)**	**Maximal score (MS)**	**Mean ±SD**	**Mean/MS (%)**
**PC1: detail information of low vision rehabilitation (27%)**	40	23.8 ± 7.5	59.5
Which of the following are considered characteristics of low vision?	3	2.2 ± 0.9	72.3
What aspects would be affected by low vision?	7	4.9 ± 1.8	69.3
Which of the following methods belong to low vision rehabilitation?	7	4.1 ± 1.9	58.4
Are you familiar with low-vision aids?	11	4.7 ± 3.0	42.3
Do you think the psychological wellbeing of low vision individuals and their families and caregivers need to pay attention to?	3	2.8 ± 0.7	92.7
Do you think training is necessary for the use of low-vision aids?	3	2.8 ± 0.6	94.3
Where do you get low vision rehabilitation-related knowledge?	6	2.4 ± 1.5	40.7
**PC2: overall impression of low vision rehabilitation (20%)**	9	5.2 ± 1.9	57.8
Do you know what is low vision rehabilitation?	3	1.8 ± 0.8	60.0
Would vision rehabilitation improve individuals' quality of life?	3	2.2 ± 0.6	72.3
Are you aware of the subsidy policies provided by China disabled persons federation?	3	1.2 ± 1.0	41.0
**PC3: understandings of the current status of low vision population (13%)**	9	3.2 ± 1.4	36.0
Where are children with severe visual impairment usually educated at?	3	1.2 ± 0.6	40.3
What do individuals with severe visual impairment usually do?	6	2.0 ± 1.0	33.8
**Cumulative (60%)**			
Total	58	32.3 ± 8.4	55.6

### Factors that affected LVR awareness

Multiple logistic regressions of sum scores of total items in the public group indicated that participants with higher education levels [OR (95% CI): vocational school, 0.5 (0.3, 0.9); bachelor's degree, 0.4 (0.2, 0.7), master's degree or above, 0.4 (0.2, 0.7)], with medical background [OR (95% CI): 0.4 (0.3, 0.5)], with good visual acuity [OR (95% CI): 0.4 (0.3, 0.7)], having eye checked every year or less [OR (95% CI): 0.4 (0.2, 0.7)] (Table 4).

**Table 4:** Multiple logistic regressions among awareness and related factors in the public^*^.

**Factors**	***N***	**Total**		PC1^**^		PC2^**^		PC3^**^	
		**OR (95% CI)**	***p*** **-Value**	**OR (95% CI)**	***p*** **-Value**	**OR (95% CI)**	***p*** **-Value**	**OR (95% CI)**	***p*** **-Value**
**Age**									
≤40 years	701	Ref		Ref		Ref		Ref	
>40 years	251	1.1 (0.8, 1.5)	0.63	1.1 (0.8, 1.5)	0.64	1.4 (1.0, 2.0)	0.05	**1.4 (1.0, 1.9)**	**0.04**
**Gender**									
Male	320	Ref		Ref		Ref		Ref	
Female	632	0.7 (0.6, 1.0)	0.06	0.8 (0.6, 1.1)	0.21	0.8 (0.6, 1.1)	0.15	0.8 (0.6, 1.1)	0.25
**Education**									
High school or below	111	Ref		Ref		Ref		Ref	
Vocational school	161	**0.5 (0.3, 0.9)**	**0.02**	**0.5 (0.3, 0.9)**	**0.02**	**0.5 (0.3, 0.9)**	**0.01**	0.9 (0.5, 1.4)	0.58
Bachelor's degree	478	**0.4 (0.2, 0.7)**	**<0.01**	**0.5 (0.3, 0.9)**	**0.01**	**0.2 (0.1, 0.3)**	**<0.01**	**0.5 (0.3, 0.9)**	**0.01**
Master's degree or above	202	**0.4 (0.2, 0.7)**	**<0.01**	**0.6 (0.3, 0.9)**	**0.03**	**0.2 (0.1, 0.4)**	**<0.01**	**0.6 (0.3, 1.0)**	**0.04**
**Region**									
Rural and township area	273	Ref		Ref		Ref		Ref	
Urban	679	0.8 (0.6, 1.1)	0.26	0.9 (0.6, 1.2)	0.37	**0.7 (0.5, 0.9)**	**0.02**	0.9 (0.7, 1.2)	0.52
**Occupation**									
Without medical background	579	Ref		Ref		Ref		Ref	
With medical background (vision not included)	373	**0.4 (0.3, 0.5)**	**<0.01**	**0.4 (0.3, 0.6)**	**<0.01**	**0.5 (0.3, 0.7)**	**<0.01**	0.9 (0.7, 1.2)	0.44
**Self-reported PVA** ^**^									
Poor vision	105	Ref		Ref		Ref		Ref	
Moderate vision	165	0.8 (0.5, 1.4)	0.41	1.3 (0.8, 2.2)	0.32	**0.5 (0.3, 0.9)**	**0.02**	**0.4 (0.3, 0.7)**	**<0.01**
Good vision	682	**0.4 (0.3, 0.7)**	**<0.01**	0.7 (0.5, 1.1)	0.15	**0.4 (0.2, 0.6)**	**<0.01**	**0.5 (0.3, 0.7)**	**<0.01**
**Frequency of eye check**									
Never	100	Ref		Ref		Ref		Ref	
Only when needed	439	0.7 (0.5, 1.2)	0.24	0.8 (0.5, 1.3)	0.41	1.3 (0.8, 2.2)	0.27	0.8 (0.5, 1.2)	0.25
Interval >1 year	188	0.6 (0.4, 1.0)	0.06	0.7 (0.4, 1.1)	0.12	**2.0 (1.1, 3.5)**	**0.02**	0.7 (0.4, 1.1)	0.11
Interval ≤ 1 year	225	**0.4 (0.2, 0.7)**	**<0.01**	**0.4 (0.2, 0.6)**	**<0.01**	**2.0 (1.1, 3.5)**	**0.02**	0.8 (0.5, 1.4)	0.44

* Regression functions were conducted four times, for total item, PC1, PC2 and PC3, respectively. Y ≥ mean score was defined as 0 (high awareness), Y < mean score was defined as 1 (low awareness). *P* < 0.05 was statistically significant. Statistically significant p-values and 95% confidence interval (95% CI) was listed in bold.

** Self-reported PVA stands for presented visual acuity of the better eye in daily life reported by the research participants themselves. PC1 stands for awareness of low vision rehabilitation. PC2 stands for awareness of low vision. PC3 stands for understanding of the current status of low vision population.

For the vision professionals, participants who aged elder than 40 years old [OR (95% CI): 0.5 (0.3, 0.9)], with a better visual acuity [OR (95% CI): moderate, 0.2 (0.0, 0.9), good, 0.1 (0.0, 0.5)] demonstrated a better awareness of low vision. Besides, vision professionals who knew the low vision referral process of the institute which he/she worked in [OR (95% CI): 0.5 (0.3, 0.9)] showed a better awareness than those who did not know (Table 5).

**Table 5:** Multiple logistic regressions awareness and related factors in the vision professionals^*^.

**Factors**	***N***	**Total**		PC1^**^		PC2^**^		PC3^**^	
		**OR (95% CI)**	***p*** **-Value**	**OR (95% CI)**	***p*** **-Value**	**OR (95% CI)**	***p*** **-Value**	**OR (95% CI)**	***p*** **-Value**
**Age**									
≤40 years	349	Ref		Ref		Ref		Ref	
>40 years	78	**0.5 (0.3, 0.9)**	**0.01**	**0.5 (0.3, 0.9)**	**0.02**	0.6 (0.4, 1.1)	0.12	0.9 (0.5, 1.5)	0.58
**Gender**									
Male	112	Ref		Ref		Ref		Ref	
Female	315	1.6 (1.0, 2.5)	0.06	1.2 (0.7, 1.9)	0.48	1.3 (0.8, 2.1)	0.35	0.6 (0.4, 1.0)	0.07
**Education**									
Vocational school or below	81	Ref		Ref		Ref		Ref	
Bachelor's degree	189	1.3 (0.7, 2.3)	0.37	0.8 (0.4, 1.4)	0.37	1.0 (0.5, 1.8)	0.87	1.8 (1.0, 3.2)	0.07
Master's degree or above	157	0.7 (0.3, 1.3)	0.25	**0.4 (0.2, 0.9)**	**0.03**	1.1 (0.5, 2.4)	0.73	1.1 (0.5, 2.3)	0.78
**Region**									
Rural and township area	39	Ref		Ref		Ref		Ref	
Urban	388	1.0 (0.5, 2.1)	0.97	1.5 (0.7, 3.3)	0.28	0.9 (0.4, 1.9)	0.72	0.7 (0.3, 1.6)	0.44
**Occupation**									
Other visual related professionals	36	Ref		Ref		Ref		Ref	
Visual technicians/assistants/nurses	131	0.8 (0.3, 1.8)	0.55	1.1 (0.5, 2.5)	0.88	1.2 (0.5, 2.8)	0.67	1.2 (0.5, 2.9)	0.63
Ophthalmologist	136	1.2 (0.5, 2.9)	0.65	1.9 (0.8, 4.6)	0.14	0.6 (0.3, 1.4)	0.26	1.3 (0.5, 3.0)	0.60
Optometrist	124	0.5 (0.2, 1.1)	0.09	0.8 (0.4, 2.0)	0.70	0.7 (0.3, 1.6)	0.40	0.9 (0.4, 2.0)	0.71
**Self-reported PVA** ^**^									
Poor vision	18	Ref		Ref		Ref		Ref	
Moderate vision	52	**0.2 (0.0, 0.9)**	**0.04**	**0.2 (0.0, 1.0)**	**0.04**	0.6 (0.2, 2.0)	0.41	1.5 (0.5, 4.7)	0.53
Good vision	357	**0.1 (0.0, 0.5)**	**<0.01**	**0.1 (0.0, 0.3)**	**<0.01**	0.9 (0.3, 2.6)	0.85	1.5 (0.5, 4.1)	0.45
**Frequency of eye check**									
Never	12	Ref		Ref		Ref		Ref	
Only when needed	145	0.9 (0.2, 3.2)	0.84	1.2 (0.3, 4.4)	0.79	3.5 (0.9, 12.8)	0.06	0.2 (0.0, 1.4)	0.10
Interval >1 year	65	0.8 (0.2, 3.0)	0.71	1.4 (0.4, 5.6)	0.61	1.3 (0.3, 5.3)	0.67	0.2 (0.0, 1.6)	0.12
Interval ≤ 1 year	205	0.8 (0.2, 2.9)	0.72	1.4 (0.4, 5.0)	0.62	1.0 (0.3, 3.7)	0.96	0.1 (0.0, 1.2)	0.07
**Institution grade**									
Other	170	Ref		Ref		Ref		Ref	
Grade three	257	0.6 (0.4, 1.0)	0.07	**0.6 (0.4, 1.0)**	**0.04**	**1.9 (1.1, 3.2)**	**0.02**	**0.6 (0.3, 0.9)**	**0.03**
**Presence of LVC** ^**^									
No, no intention to establish	36	Ref		Ref		Ref		Ref	
No, but intend to establish one	81	1.6 (0.7, 3.7)	0.29	2.3 (0.9, 5.5)	0.07	0.4 (0.1, 1.1)	0.09	1.8 (0.8, 4.2)	0.18
Yes, immature conditions/technology	81	1.5 (0.6, 3.7)	0.38	1.6 (0.6, 4.2)	0.33	0.4 (0.1, 1.2)	0.09	1.9 (0.8, 4.8)	0.16
Yes, mature conditions/technology	229	2.4 (1.0, 6.0)	0.05	**2.9 (1.1, 7.5)**	**0.03**	**0.2 (0.1, 0.6)**	**<0.01**	**2.6 (1.1, 6.3)**	**0.04**
**Presence of LVR referral process** ^**^									
Don't know/no	119	Ref		Ref		Ref		Ref	
Yes	308	**0.5 (0.3, 0.9)**	**0.03**	0.7 (0.4, 1.2)	0.17	**0.4 (0.2, 0.7)**	**<0.01**	0.7 (0.4, 1.3)	0.31

* Regression functions were conducted four times, for total item, PC1, PC2 and PC3, respectively. Y ≥ mean score was defined as 0 (high awareness), Y < mean score was defined as 1 (low awareness). *P* < 0.05 was statistically significant. Statistically significant p-values and 95% confidence interval (95% CI) was listed in bold.

** Self-reported PVA stands for presented visual acuity of the better eye in daily life reported by the research participants themselves. PC1 stands for detail information of low vision rehabilitation. PC2 stands for overall impression of low vision rehabilitation. PC3 stands for understanding of the current status of low vision population. LVC stands for low vision center. LVRRP stands for low vision rehabilitation referral process.

### Sources of knowledge on low vision rehabilitation

**Figure 4:**
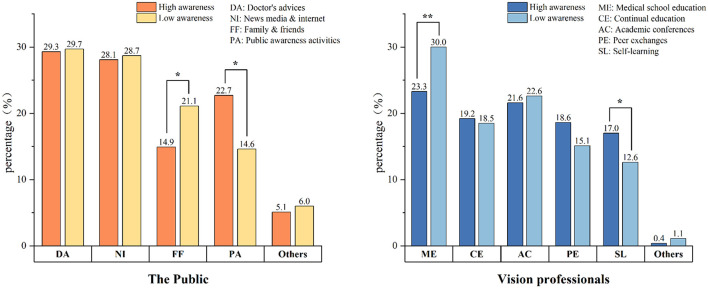
Sources of knowledge on low vision rehabilitation. High awareness stands for an individual's total score higher than mean score. Low awareness stands for an individual's score lower than mean score. *p* < 0.05 was statistically significant. *p < 0.05. **p < 0.01.

The sum score of total items above the average was defined as high awareness and below the average was defined as low awareness. For the public, the main sources of obtaining LVR information were through doctors' advice (about 29%) and news media or internet (about 28%). A higher percentage of participants obtain parts of the knowledge from public awareness activities in the high awareness group, compared to the low awareness group (22.7 vs. 14.6%, *p* < 0.05). For the vision professionals, the main sources of obtaining LVR information in the low awareness group was through medical school education (30.0%) while in the higher awareness group, knowledge pathways were pluralistic, including medical school education, continual education, conference, and peer exchanges. A higher percentage of participants obtain parts of the knowledge from self-learning in the high awareness group compared to the low awareness group (17.0 vs. 12.6%, *p* < 0.05, Figure 4).

## Discussion

We demonstrate that both the public and vision professionals lack overall awareness of LVR in China through this study. Misconceptions and the lack of awareness about rehabilitation services among people pose a significant barrier to the development of LVR (19–23). It is essential to enhance the importance of low vision publicity, then people can understand and pay more attention to it.

### Low awareness of LVR in both the public and vision professionals

The mean score of total items was only one third of the maximal score in the public. The low awareness of LVR was not only demonstrated in low cognition of what is LVR, but also manifested in less attention to the living status of people with low vision. The public with higher levels of education and having medical background demonstrated better awareness. It is no doubts that people working in medical system have a better cognition of health compared to the public. Participants with better education have a higher possibility to represent a better level of cognition (24–27), or have better learning abilities, which leads to effective pathways to obtain LVR-related knowledge (28).

The average score of total items in the vision professionals is about one half of the maximal score, which is far from enough for a qualified vision professional. Most vision professionals know what low vision is and believe that visual rehabilitation training is necessary, while lack the awareness on how to deliver LVR. Vision professionals also show low attention to the survival status of individuals with low vision. Similar results have been presented in previous studies (29, 30). Research by Jose indicated that 64.0% of vision professionals lack awareness of LVR, and 62.1% of the vision professionals lack relevant training, which is a major obstacle to providing LVR services (31).

It is surprised to found that a better awareness of LVR of a vision professional was not related to whether an institution had a visual rehabilitation center or not, but associated with a settled process of low vision referral. We speculated that referral process indicated that this institution has a high possibility of implementing LVR services. Even if a doctor did not deliver LVR themselves, a timely referral informed a low vision person where to get LVR services, was equally helpful (32). The presence of a referral process allows resources and information sharing among staff, therefore, they would have a better understanding of LVR. This speculation is supported by literature researches. Data showed that the level of a hospital is not determined by department settings, but relies on high quality services (33, 34).

### To improve awareness of the public

**Figure 5:**
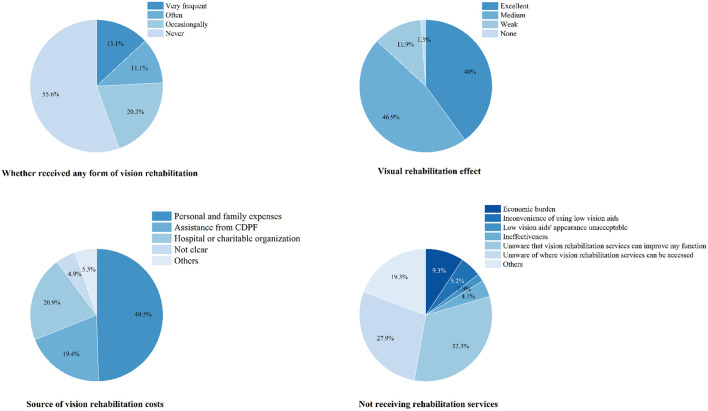
Low vision rehabilitation state of individuals with visual impairment.

Although the public awareness on detailed LVR is limited at this moment, participants with visual impairments who have received rehabilitation services show a positive attitude toward visual rehabilitation (Figure 5), which is consistent with previous findings (4, 35–37).

The study also showed that the public mainly obtain low vision rehabilitation information through doctors' advice and public media. This emphasizes that the first visit doctor delivers low vision rehabilitation advice to the patient is important (38). The truth that participants who undergone eye check more frequently shew better awareness could also demonstrate the statement. The second method revealed form the results was that vision professionals should increase positive guidance and scientific education of LVR though public media. A survey conducted in Australia concerning LVR services revealed that 85% of Australian participants stated that receiving information about LVR was a facilitating factor in utilizing low vision services (32). However, negative behaviors and attitudes from ophthalmologists in introducing and recommending LVR services to patients have been found to be barriers to patients' acceptance of LVR services (32, 39).

In summary, public awareness can be gradually influenced (40). Doctors' advice in the clinic and scientific education though public media would help to improve the situation.

### To improve awareness of vision professionals

The primary approach of obtaining information was through studying during medical school education, for vision professionals with low awareness of LVR. For vision professionals with higher awareness, they get their knowledge from diverse ways, such as medical school education, continual education, academic conferences, and peer exchanges.

The results revealed that to improve basic low vision knowledge and awareness, college or university education would be helpful. Besides, university and college time is crucial for personal growth and the reshaping of values (41, 42). Low vision education during this period would help to create a systematic impression and be much more efficient than later casual learning (43).

Comprehensive and cutting-edge knowledge of low vision would mainly come from literatures and academic discussions. And standard LVR procedures would be provided in continual education courses (44). The cultivation of a better awareness and abilities in the later stage, attending more low vision conferences, symposiums and continual education would be a second chance.

### Limitations

The participants of the present study covered various provinces and cities nationwide in China, but with a primary concentration in eastern China and the hospitals involved were relatively in a high level in China. As the eastern side is much more developed than the northwest and southwest of China, if the awareness of the eastern area was not sufficient, then other areas would be even lower. The study might overestimate the current awareness of the public and the vision professional in China, but would not impact the solutions it revealed. Another limitation of the study comes from a cross-sectional study with self-reported answers, which can't tell why the study subjects lack awareness as well as the quantitative effectiveness the solutions, which needs later longitudinal studies to confirm.

## Conclusions

The awareness of LVR in China is insufficient in both the public and vision professionals. Emphasizing the importance of doctor's advices in the clinic and scientific education through public media to improve the awareness of the public. And it is crucial to develop low vision education in medical curriculum and expand low vision conferences and continuing education to improving awareness of the vision professionals.

## Data Availability

The original contributions presented in the study are included in the article/supplementary material, further inquiries can be directed to the corresponding author.
