# Supplementary material for: Public awareness of low vision rehabilitation in China
Source: Front Med (Lausanne). 2025 Aug 22;12:1659439. doi: 10.3389/fmed.2025.1659439 (PMC12411518; doi:10.3389/fmed.2025.1659439)
Supplement: Supplementary file 1 [file Data_Sheet_1.docx]

Appendix 1. Items of the public awareness survey of low vision rehabilitation in China

**General questions for all**

1. How old are you?
2. ≤40 years
3. >40 years

1. What is your gender?
2. Male
3. Female

1. What is your education level?
2. High school or below
3. Vocational school
4. Bachelor's degree
5. Master's degree or above

1. Where do you live?
2. Rural and suburban area
3. Urban area

1. How often do you go to the hospital for an eye check?
2. Never
3. Only when needed
4. Interval >1 year
5. Interval ≤1 year

1. Where are children with severe visual impairment usually educated at? (Multiple choices allowed)
2. Ordinary school
3. Blind school/special education school
4. Drop out of school
5. Don't know

1. What do individuals with severe visual impairment usually do for a living? (Multiple choices allowed)
2. Massage therapist
3. Tuner
4. Teacher
5. Farmer
6. Unemployed
7. Others

1. What is your occupation (Choose B/C to continue. Choose A to go to B1)
2. Ophthalmologist/Optometrist/Visual technicians/nurses/Other visual related professionals
3. Medical professionals (vision professionals not included)
4. Non-medical professionals

**Questions for the public**

A1. Which of the following are considered as low vision? (Multiple choices allowed)

1. poor vision
2. Need glasses
3. Amblyopia, nearsightedness, and glaucoma are low vision
4. decreased visual acuity that cannot be improved by glasses, surgery, or medicine
5. Severely contracted visual range
6. Uncertainty
7. Others

A2. Does low vision affect individuals’ lives and work? (Choose A to continue. Choose B/C to go to A4)

1. Yes
2. No
3. Not sure

A3. What aspects would be affected by low vision? (Multiple choices allowed)

1. Mobility
2. Reading/writing
3. Personal hygiene
4. Normal work
5. entertainment and social activities
6. Mental health
7. Unclear
8. Others

A4. How much do you know about low vision rehabilitation? (Choose A/B/C to continue, choose D go to A10)

1. Very familiar
2. General understanding
3. Know a little bit
4. Know nothing at all, never heard of it

A5. Where do you get low vision rehabilitation-related knowledge? (Multiple choices allowed)

1. Doctor's advice
2. News media, internet
3. Family & friends
4. Public awareness activities
5. Others

A6. Would low vision rehabilitation improve individuals’ quality of life?

1. Definitely can
2. To a great extent can
3. To a great extent can’t
4. Definitely can’t

A7. Which of the following methods belong to low vision rehabilitation? (Multiple choices allowed)

1. Glasses
2. Low vision aids (telescopes, magnifying glasses, electronic visual aids and other kinds of aids)
3. Visual function training
4. Skills training
5. Improvement of environments
6. Psychological counseling
7. Not sure
8. Others

A8. Which places provide low vision rehabilitation services? (Multiple answers allowed)

1. Hospitals
2. Optometric centers
3. Low vision training institutions
4. China Disabled Persons' Federation or Disabled Persons' Federations in different areas.
5. Special education schools
6. Glasses stores
7. Not sure
8. Others

A9. Are you aware of the subsidy policies provided by China Disabled Persons Federation?

1. Very familiar
2. General understanding
3. Know a little bit
4. Know nothing at all, never heard of it

A10. What is the visual acuity of your better eye? (if you wear glasses, please provide the vision with glasses) (Choose A/B/F go to C1, otherwise the end)

1. Cannot see the top row of the visual acuity chart
2. Can see the top rows clearly
3. Can see the middle or lower rows clearly
4. Can see well, can see the penultimate rows clearly
5. Don’t know, but can see clearly
6. Don’t know, but cannot see clearly

The following questions should be answered by vision professionals.

B1. What is your occupation?

1. Ophthalmologist
2. Optometrist
3. Visual technicians, assistants, and nurses
4. Other vision-related professionals

B2. What is the grade of your hospital & rehabilitation institution?

1. Third-grade
2. Others

B3. Does your organization have a low vision center/visual rehabilitation department?

1. Yes, conditions/technology are mature
2. Yes, but conditions/technology are immature
3. No, but there is an intention to establish one
4. No, and there is no intention to establish one
5. Not appropriate for me to answer

B4. Does your organization have a referral process for low-vision rehabilitation?

1. No / do not know if there is a referral process
2. Yes
3. Not appropriate for me to answer

B5. Which of the following are considered characteristics of low vision? (Multiple choices allowed)

1. Amblyopia
2. Any disease that results in visual impairment that cannot be improved
3. Any disease that results in vision that cannot be improved（BCVA better than 0.05 less than or equal to 0.3）
4. Visual field radius less than 10 °
5. Unsure

B6. Do you think low vision may affect your life and work? (Choose A to continue, choose B/C to go to B8)

1. Yes
2. No
3. Not sure

B7. What impacts would low vision have? (Multiple choices allowed)

1. Mobility
2. Reading/writing
3. Personal hygiene
4. Normal work
5. Entertainment and social activities
6. Psychological status
7. Unknown
8. Others

B8. Do you know what is low vision rehabilitation? (Choose A/B/C to continue, choose D go to B16)

1. Very familiar
2. General understanding
3. Know a little bit
4. Know nothing at all, never heard of it

B9. Where do you get low vision rehabilitation-related knowledge? (Multiple choices allowed)

1. Medical school education
2. Continual education
3. Academic conferences
4. Peer exchanges
5. Self-learning
6. Others

B10. Which of the following methods belong to low vision rehabilitation? (Multiple choices allowed)

1. Glasses
2. Low vision aids
3. Visual function training
4. Skill rehabilitation training
5. Improvement of environments
6. Psychological counseling
7. Not sure
8. Others

B11. Which of the following low-vision aids are you familiar with? (Multiple choices allowed)

1. Table lamps and caps
2. Guide dogs and canes
3. Ultrasound guides and GPS
4. Smart glasses and smart phones
5. Sensors and sound devices
6. Reading frames
7. Various kinds of markers
8. Large print books or tools
9. Mirrors
10. Prism
11. Do not know
12. Others

B12. Do you think training is necessary for the use of low-vision aids?

1. Professional training is needed
2. No, people can study and learn how to use it by themselves or by their family
3. No, it is simple and easy to use
4. Don't know

B13. Do you think the psychological well-being of low vision individuals and their families and caregivers need to pay attention to?

1. Both need to pay attention to
2. Low vision individuals need it, families & caregivers don’t
3. Neither
4. Never think about it

B14. Would vision rehabilitation improve individuals’ quality of life?

1. Definitely can
2. To a great extent can
3. To a great extent can’t
4. Definitely can’t

B15. Are you aware of the subsidy policies provided by Disabled Persons’ Federation?

1. Very familiar
2. General understanding
3. Know a little bit
4. Know nothing at all, never heard of it

B16. What’s your visual acuity of the better eye in daily life? (if you wear glasses, please provide the vision with glasses) (Choose B/C/E go to C1, otherwise the end)

1. 0.3 (4.5) and above
2. Worse than 0.3 (4.5)
3. Only the upper lines of the letters on the visual acuity chart can be seen
4. Don’t know, but can see clearly in daily life
5. Don’t know, but cannot see clearly in daily life

The following questions should be answered by persons with severe visual impairment

C1. Have you received any form of visual rehabilitation? (Choose A/B/C to continue, choose D go to C4)

1. Yes, very frequently
2. Yes, often
3. Yes, occasionally
4. No, never

C2. What are your opinions on visual rehabilitation?

1. Excellent
2. Medium
3. Weak
4. None

C3. How is your visual rehabilitation payment covered? (Multiple choices allowed)

1. Personal and family expenses
2. Subsidies from the Disabled Persons' Federation
3. Assistance from hospitals or charitable organizations
4. Not clear
5. Others

C4. Why haven’t you received visual rehabilitation services? (Multiple answers allowed)

1. Economic burden
2. Inconvenience of using low vision aids
3. Unacceptable of the appearance of low vision aids
4. Inefficiency
5. Unaware of vision rehabilitation services can improve visual function
6. Unaware of where vision rehabilitation services can be obtained
7. Others
